# NRF2 is essential for adaptative browning of white adipocytes

**DOI:** 10.1016/j.redox.2023.102951

**Published:** 2023-10-31

**Authors:** Marco Bauzá-Thorbrügge, Eduard Peris, Shabnam Zamani, Peter Micallef, Alexandra Paul, Stefano Bartesaghi, Anna Benrick, Ingrid Wernstedt Asterholm

**Affiliations:** a Department of Physiology/Metabolic Physiology, Institute of Neuroscience and Physiology, The Sahlgrenska Academy at University of Gothenburg, Gothenburg, Sweden; b Department of Biology and Biological Engineering, Division of Chemical Biology, Chalmers University of Technology, Gothenburg, Sweden; c Bioscience Metabolism, Research and Early Development Cardiovascular, Renal and Metabolism, BioPharmaceuticals R&D, AstraZeneca, Gothenburg, Sweden; d School of Health Sciences, University of Skövde, Skövde, Sweden; e The Department of Biomedical Engineering, University of Texas at Austin, Austin, TX, USA

**Keywords:** Adipose tissue, Redox stress, NRF2, N-acetylcysteine, Lactate

## Abstract

White adipose tissue browning, defined by accelerated mitochondrial metabolism and biogenesis, is considered a promising mean to treat or prevent obesity-associated metabolic disturbances. We hypothesize that redox stress acutely leads to increased production of reactive oxygen species (ROS), which activate electrophile sensor nuclear factor erythroid 2-Related Factor 2 (NRF2) that over time results in an adaptive adipose tissue browning process. To test this, we have exploited adipocyte-specific NRF2 knockout mice and cultured adipocytes and analyzed time- and dose-dependent effect of NAC and lactate treatment on antioxidant expression and browning-like processes.

We found that short-term antioxidant treatment with N-acetylcysteine (NAC) induced reductive stress as evident from increased intracellular NADH levels, increased ROS-production, reduced oxygen consumption rate (OCR), and increased NRF2 levels in white adipocytes. In contrast, and in line with our hypothesis, longer-term NAC treatment led to a NRF2-dependent browning response. Lactate treatment elicited similar effects as NAC, and mechanistically, these NRF2-dependent adipocyte browning responses *in vitro* were mediated by increased heme oxygenase-1 (HMOX1) activity. Moreover, this NRF2-HMOX1 axis was also important for β3-adrenergic receptor activation-induced adipose tissue browning *in vivo.*

In conclusion, our findings show that administration of exogenous antioxidants can affect biological function not solely through ROS neutralization, but also through reductive stress. We also demonstrate that NRF2 is essential for white adipose tissue browning processes.

## Highlights

• N-acetylcysteine or lactate both induce reductive stress in white adipocytes.
• Reductive stress induces white adipocyte browning via a NRF2-dependent mechanism.
• β3-adrenergic receptor activation-induced browning is partly mediated by NRF2.
• NRF2-dependent white adipocyte browning is mediated by heme oxygenase-1 activity.

## Graphical abstract

**Figure:**
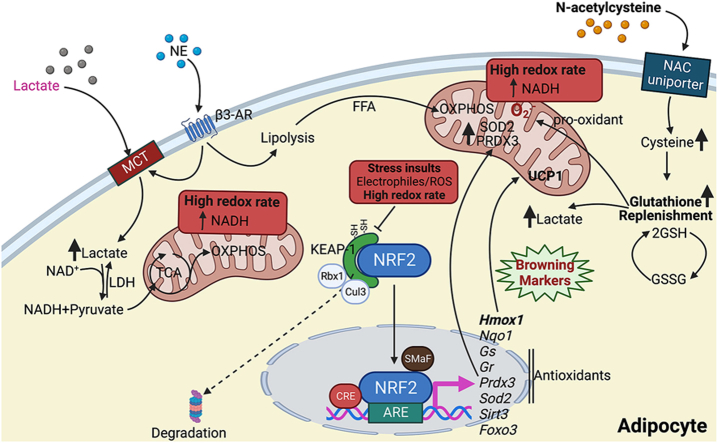

## 1 Introduction

Chronic overnutrition increases the risk of insulin resistance and type-2 diabetes. Healthy adipose tissue expansion protects against the deleterious effect of overnutrition by acting as a “metabolic sink” that removes excess nutrients from the bloodstream. However, the capacity for healthy adipose tissue expansion is often reduced in obesity. A key question in the field of metabolic research is therefore whether and how dysfunctional adipose tissue can be transformed into healthier adipose tissue. In this regard, adipose tissue browning as defined by increased mitochondrial metabolism and biogenesis is considered a promising strategy that accelerates nutrient uptake and thermogenesis. Adipose tissue browning can be triggered by chronic exposure by lipolysis-inducing stressors such as cold temperature, β-adrenergic receptor (AR)-activation, and by some tumor-derived factors [[1], [2], [3], [4], [5]]. Moreover, redox stress induced by oxidants [6], metabolites such as lactate and β-hydroxybutyrate [7], and elevated mitochondrial reactive oxygen species (ROS) levels [8,9] can also lead to browning with increased expression of uncoupling protein-1 (UCP1) that presumably alleviates the redox pressure [7,8,10]. In this area, we have recently demonstrated that acute inhibition of the Ca^2+^ pump SERCA increases the ROS production associated with reduced oxygen consumption rate (OCR) while genetic ablation of SERCA2 leads to elevated UCP1 levels and accelerated mitochondrial metabolism in white adipocytes [11]. Thus, adipose tissue browning can be referred to as a mitohormesis process, i.e. a coordinated adaptive response to metabolic stress leaving the cell less susceptible to subsequent perturbations. However, numerous studies show that chronically elevated ROS levels are involved in the pathogenesis of metabolic diseases rather than stimulating mitohormesis. Therefore, it has been a growing interest in attenuating oxidative stress by stimulating the endogenous antioxidant system or supplementing the diet with exogenous antioxidants. Among these antioxidants, N-acetylcysteine (NAC) have been proposed to protect cells from oxidative stress [[12], [13], [14]] and to reduce lipid accumulation and insulin resistance in high-fat diet (HFD)-fed mice [[15], [16], [17], [18]]. NAC is metabolized to l-cysteine, a precursor to reduced glutathione [19]. Glutathione neutralizes ROS and nitrogen species through both direct and indirect scavenging catalyzed by glutathione peroxidase in a NADH–dependent reaction [20]. However, too high levels of reducing equivalents (NADH, NADPH, and glutathione) can lead to reductive stress, a concept that was first defined by Gores et al., in 1989 [21,22]. While reductive stress is the opposite of oxidative stress, both states can trigger increased ROS production. For instance, increased glycolysis and TCA cycle rates in hyperglycemic conditions lead to NADH overproduction and NADH-mediated electron pressure can increase the superoxide formation from the mitochondrial electron transport chain (ETC) via reduction of molecular oxygen to superoxide [[22], [23], [24]]. Accordingly, we have shown that short-term NAC treatment leads to reductive stress in cultured adipocytes and adipose tissue in mice as judged by enhanced β3-AR activation-induced mitochondrial ROS production, reduced OCR, reduce β3-AR-activation-induced browning and enhanced stabilization of Nuclear factor erythroid 2-related factor 2 (NRF2) [25]. NRF2, through KEAP1, is a sensor for ROS and electrophiles that increases the transcription of cytoprotective antioxidation-related genes to that way maintain intracellular redox homeostasis [[26], [27], [28]]. Interestingly, NRF2 also supports mitochondrial metabolism and biogenesis [29]. Based on all these observations, we hypothesize that increased ROS levels in NAC-treated white adipocytes trigger an adaptive decrease in 10.13039/100010257OCR along with increased NRF2-activation that in turn increases the expression of endogenous antioxidant enzymes and eventually leads to mitohormesis. To test this hypothesis, we have exploited adipocyte-specific NRF2 knockout mice and cultured adipocytes and analyzed time- and dose-dependent effect of NAC and lactate (that also triggers reductive stress [7]) on antioxidant expression and browning-like processes. We have also investigated the role of NRF2 in conventional β3-AR-activation induced adipose tissue browning.

## 2 Material and methods

### 2.1 Adipocyte culture

3T3-L1 adipocytes (ZenBio, NC, USA) were differentiated and cultured as previously described [25]. The stromal vascular fraction (SVF) of mouse inguinal white adipose tissue (IWAT) was differentiated into mature adipocytes as in Ref. [25], but with the addition of 1 μM rosiglitazone (1 μM, Sigma, R2408) in the first differentiation step. Studies were performed in mature adipocytes (based on occurrence of large lipid droplets) between days 9–10 from the start of differentiation. The adipocytes were incubated with or without N-acetyl-l-cysteine (1 mM, Sigma A9165), sodium l-lactate (25 mM, Sigma, L7022), D-l-Sulforophane (1 μM, Sigma, S4441), and zinc protoporphyrin (ZnPP, 5 μM, Tocris, 0746), and the medium was changed every two days. For the induction of beige-like adipocytes, 3T3-L1 cells were treated with Rosiglitazone (1 μM) + Triiodothyronine (T3, 50 nM, Sigma, T6397) as described by Asano et al. [30].

### 2.2 In vitro silencing of HMOX-1

3T3-L1 preadipocytes were seeded in 12-well plates. Upon reaching confluence, cells were induced to differentiate as described above. On day 5 of differentiation, the cells were transiently transfected with 5 nM siRNA (Hmox1 siRNA ID 158978 or siRNA Silencer™ Negative Control No. 1-AM4611, Invitrogen) using Lipofectamine 3000 (2.25 μl/well; Invitrogen) according to the manufacturer's instructions. Two days after transfection, cells were treated with lactate or NAC. For the 1-week treatment, NAC and transfection were refreshed every two days to achieve persistent inhibition of HMOX1. The cells were harvested for RNA or protein extraction at the indicated time points.

### 2.3 Animal experiments

5-week-old male wild-type C57BL/6J mice were obtained from Charles River (Freiburg, Germany) and housed under standard conditions. LoxP flanked NRF2 knock-in mice (C57BL; 6-Nfe2l2tm1.1Sred/SbisJ/J stock 025433, Jackson Laboratory) were crossed with adiponectin promoter driven Cre transgenic mice (B6; FVB-Tg (Adipoq-cre)1Evdr/J stock 010803, Jackson Laboratory), both on a C57BL/6 background, to selectively knock-out NRF2 from adipocytes. Littermates not expressing Cre were used as wild-type (WT) controls. Water and food (Teklad Global 16 % Protein Rodent Diet, Harlan, Madison, WI, USA) were provided *ad libitum*. High-fat diet (HFD) (60 % kcal from fat: D12492, Research Diets Inc.) was provided *ad libitum* for 12 weeks as indicated. All experiments were performed at the laboratory of Experimental Biomedicine and were approved by the Regional Ethical Committee in Gothenburg.

### 2.4 In vivo antioxidant treatment

6-week-old C57BL/6J male mice on regular chow (Teklad Global 16 % Protein Rodent Diet, Harlan, Madison, WI, USA) *ad libitum* were randomly divided into control and antioxidant treatment groups. The antioxidant group received NAC through their drinking water (0.5, 2 or 10 g/L, Sigma A9165) for the indicated time periods. At the end of the experiment, animals were euthanized, blood was collected, and tissues were rapidly dissected, weighed and snap-frozen in liquid nitrogen or processed right away. Serum and tissue samples were kept at −80 °C until further analysis.

### 2.5 In vivo induction of browning of white adipose tissue by the β3 agonist CL-316,243 or lactate

9-week-old C57BL/6J male mice on regular chow *ad libitum* were daily injected IP for 10 days with sterile vehicle (phosphate buffered saline (pH 7.4)), CL-316,243 (1 mg/kg, Sigma Aldrich, USA), or sodium l-lactate (2 g/kg, Sigma Aldrich, USA). Final solutions for injections were filtered through sterile 0.2 μm syringe filter unit. Mice were sacrificed on day 11.

### 2.6 In vivo inhibition of Hemoxigenase 1 (HMOX1)

7-week-old C57BL/6J male mice received daily IP injection of sterile vehicle (phosphate buffered saline, pH 7.4), CL-316,243 (1 mg/kg, Sigma Aldrich, USA), zinc protoporphyrin (ZnPP) (250 μg/kg, MedChemExpress, USA), or a combination of CL-316,243 and ZnPP for 10 days. Prior to injection, the final solutions were filtered through a sterile 0.2 μm syringe filter unit. The mice were sacrificed on the 11th day.

### 2.7 Adipocyte isolation and culture

Inguinal white adipose tissue (IWAT) was isolated from 8-week-old C57BL/6J mice to obtain primary adipocytes. The tissue samples were minced and digested using collagenase type II at a concentration of 1 mg/mL for 45–60 min at 37 °C. The digested tissue was then passed through a 100-mm nylon mesh to separate the floating adipocytes. The adipocytes were washed with KrebsRinger glucose buffer containing 1 % BSA. Adipocytes were incubated in a membrane mature adipocyte aggregate culture as described in Ref. [31] to remain functional. After 72h of treatment with vehicle or 1 mM NAC, adipocytes were collected for analysis.

### 2.8 Gene expression analysis

Total RNA was isolated from 3T3-L1 adipocytes and IWAT using Isol-RNA lysis reagent (5PRIME, Germany) and ReliaPrep RNA Miniprep System (Promega, WI, USA). RNA concentration was determined with a Nanodrop 1000 spectrophotometer (ThermoFisher Scientific). RNA was reversely transcribed into cDNA using the qScript kit (Quanta BioSciences, MD, USA). Expression of mRNA was determined on a QuantStudio 7 system using SYBR Green Master Mix (Applied Biosystems, MA, USA), and quantified using the 2^−ΔΔCt^ method with *β-actin* or *Gapdh* as the endogenous control [32]. Primer sequences are shown in Suppl. Table 1.

### 2.9 Protein expression quantification

IWAT (whole tissue and isolated adipocytes in culture as described in Ref. [33]) was extracted with lysis buffer using 5 mm stainless steel beads and a TissueLyser II (Qiagen). Cytoplasmic and nuclear protein fractions were isolated following the NE-PER™ Nuclear and Cytoplasmic Extraction Reagents (ThermoFisher, CA, USA) manufacturers’ instructions. After protein quantification assay (Pierce BCA assay, ThermoFisher, CA, USA), supernatants or cytosolic and nuclear fractions were further diluted with lysis buffer and mixed with 4x loading buffer with β-mercaptoethanol and boiled for 5 min. Samples were loaded into an AnykD Mini or midi-PROTEAN TGX Precast Protein Stainfree Gel (BioRad, CA, USA), and transferred to a blot using a Trans-Blot Turbo PVDF (polyvinylidene difluoride) Transfer Pack (BioRad). The following antibodies were used for immunoblotting: anti-PRDX3 (1:500; ab73349; Abcam), anti-SOD2 (1:5000; ab13533; Abcam), anti-NRF2 (1:5000; ab62352; Abcam), Total OXPHOS Rodent WB Antibody Cocktail (1:250; ab110413; Abcam), anti-UCP1 (1:500 dilution; ab10983; Abcam), anti-PGC1a (1:500 dilution; ab 54481; Abcam), anti-HSP60 (1:1000 dilution; ab190828; Abcam), anti-HMOX1 (1:1000 dilution; MA1-112; Invitrogen), anti-Histone H3 (1:1000 dilution; ab1791; Abcam), anti-GAPDH (1:2000 dilution; ab8245; Abcam), anti-betaTubulin (1:1000 dilution; 2128; Cell signaling), anti-Rabbit-HRP (1:5000; #31461; ThermoFisher) and anti-Mouse-HRP (1:1000; ab6789; Abcam). Pierce Reversible Protein Stain Kit for PVDF Membranes (Pierce BCA assay, ThermoFisher, CA, USA) was used to visualize the total protein loaded. The blot pictures were acquired and analyzed with the ChemiDoc Touch imager (BioRad).

### 2.10 Mitochondria isolation

IWAT mitochondria were isolated from male mice following a protocol based on a previously reported method [34]. In brief, the fat pads were minced in 15 mL ice-cold isolation buffer (animals were pooled as necessary to achieve 0.5–1 g of sample). All subsequent steps of the preparation were performed on ice. The tissue was disrupted using a drill-driven Teflon glass homogenizer with 4–5 strokes. Homogenates were centrifuged at 800 g for 10 min at 4 °C. The supernatant was transferred into a new tube and centrifuged again at 800 g for 10 min. The resulting supernatant was transferred to a new tube and centrifuged at 8000 g for 10 min at 4 °C. After the removal of the light mitochondrial layer, the pellet was resuspended in 5 mL of isolation buffer, and the centrifugation was repeated. The final pellet was resuspended in 100 μL of isolation buffer without BSA. Total protein (μg/μL) was determined using Bradford Assay reagent (Bio-Rad) and typically, 600 μg of mitochondria was obtained per IWAT sample.

### 2.11 Oxygen consumption rate

Mitochondria were seeded into XF96 Microplates (Seahorse Bioscience, Agilent Technologies, CA, USA) at a density of 0.25 μg/μL per well. Basal respiration was determined as oxygen consumption rate (OCR) in the presence of substrates alone (5 mM succinate plus 2 μM rotenone). Results were normalized to μg of mitochondrial protein in each well. Before data analysis, non-mitochondrial respiration (remaining OCR after the addition of Antimycin A) was subtracted from all individual values. ADP absolute refers to the OCR after addition of ADP, proton leak equals the OCR after the addition of oligomycin (in presence of ADP) and ATP-linked respiration is the difference between ADP absolute and proton leak OCR. Basal OCR of whole IWAT were measured in a similar manner in XF24 Microplates. About 2 mg tissue per well was used and results were normalized to mg of tissue. OCR in cultured adipocytes were measured and calculated as previously described [25]. Briefly, basal respiration was determined as OCR in the presence of substrate alone and OXPHOS parameters were calculated following Agilent instructions and conditions are described for every experiment. Results were normalized to the amount of protein (quantified with Pierce BCA assay) in each well.

### 2.12 Oral glucose tolerance test

Mice were given an oral load of glucose (1.5 g/kg body weight) after 4 h fasting. Blood samples were taken at 0, 15, 30, 60, and 120 min, and the glucose concentrations were measured using a glucometer (Bayer, Germany).

### 2.13 Serum insulin levels

Blood samples were collected from mice fasted for 4 h, and serum insulin levels were measured using a commercial ELISA according to the manufacturer's instruction (Mercodia, Sweden).

### 2.14 Body composition

Bone, fat, and lean mass were determined using the PIXImus II densitometer (Madison, WI, USA, software version 2.00). Mice were anesthetized during the procedure using Isoflurane. Calibration of the instrument was conducted as previously described [35].

### 2.15 Analyses of size, lipid, and mitochondrial content in individual adipocytes within fresh adipose tissue

These analyses were done as described previously [25]. In brief, adipose tissue was cut into <1 mm^3^ pieces for two-photon fluorescence and Coherent Anti-Stokes Raman Scattering (CARS) microscopy. To visualize living mitochondria, the samples were stained with Rhodamine 123 (Life technologies). To visualize lipids, 2 ps-pulsed laser beams at wavelengths of 817 nm and 1064 nm to probe specific vibrations in the carbon-hydrogen bonds of triglycerides. The 1064 nm beam was generated by a diode-pumped solid-state laser, while the 817 nm beam was generated by an optical parametric oscillator. These laser beams were combined and focused onto the sample using an oil immersion objective (Eclipse TE2000-E with C2 confocal scanning head, Nikon). The forward-scattered CARS signal and backscattered two-photon fluorescence signal were recorded using single-photon counting detector technology and the specific vibration at 2845 cm^−1^ of carbon-hydrogen bonds in the alkylic chains of triglycerides were probed in a CARS process by overlapping 2 ps-pulsed laser beams at wavelengths 817 nm and 1064 nm (7ps, 76 MHz respectively). Multiple planes, each consisting of 512 x 512 pixels, were recorded using a dwell time of 5.04 μs per pixel to generate 3D images of the tissues. These images were analyzed by Image J using an adapted version of the quantitative voxel analysis as described previously [36], and single cells were selected manually. In this analysis, cell size, lipid- and mitochondrial content were estimated from thresholded images of the 2-photon fluorescence and CARS signals.

### 2.16 ROS production

Cellular and mitochondrial ROS production was detected using an H2DCFDA reagent or MitoSOX Red superoxide indicator (ThermoFisher, CA, USA) according to the manufacturer's instructions. In brief, after completing NAC or lactate treatment for the indicated periods of time (0, 1, 3, 6, 12, 24, 72, 168 h), cells were labeled with either H2DCFDA for 60 min or MitoSOX for 10 min. Cell nuclei were stained with Hoechst 33342 (Sigma Aldrich, USA). H2DCFDA fluorescence was quantified as one single measurement, while MitoSOX fluorescence was quantified as area under the curve (AUC) from 30-min recordings.

### 2.17 UCP-1 immunohistochemistry

Adipose tissue was fixed in 4 % paraformaldehyde for 24 h. After paraffin embedding and sectioning (5 μm), tissues were deparaffinized and stained as previously [11]. In brief, heat-induced antigen retrieval was performed with sodium citrate buffer (10 mM sodium citrate, 0.05 % Tween 20, pH 6.0) followed by blocking with 10 % Donkey serum containing 1 % BSA in TBST (pH 7.6) for 1 h at room temperature. Thereafter, sections were incubated overnight at 4 °C with primary antibody to mouse Ucp1 (Abcam, ab10893, 1:250), followed by 1h-incubation with secondary HRP-conjugated antibody at room temperature in the dark. Sections were counterstained with hematoxylin.

### 2.18 Intracellular NAD^+^ and NADH levels

Concentrations of NAD^+^ and NADH were measured following EnzyChrom™ NAD/NADH Assay Kit according to the manufacturer's instructions.

### 2.19 IWAT glucose uptake

D-[U–^14^C]-glucose (5 μCi/mouse, PerkinElmer, Boston, MA, USA) dissolved in PBS were given to 4 h fasted mice by gavage (300 μl/mouse). After 30 min, IWAT was dissected and analyzed as previously described [37]. Results are expressed as fraction (%) of the injected ^14^C counts per min (CPM) per mg tissue.

### 2.20 Statistical analysis

GraphPad Prism 9 (GraphPad Software, CA, USA) was used for statistical analysis. Values are shown as means ± SEM. Comparisons were performed using one-way or two-way ANOVA, or two-tailed *t*-test and p < 0.05 was considered statistically significant.

## 3 Results

### 3.1 NAC and lactate, both induce reductive stress associated with time-dependent increase in adipocyte browning

**Fig. 1:**
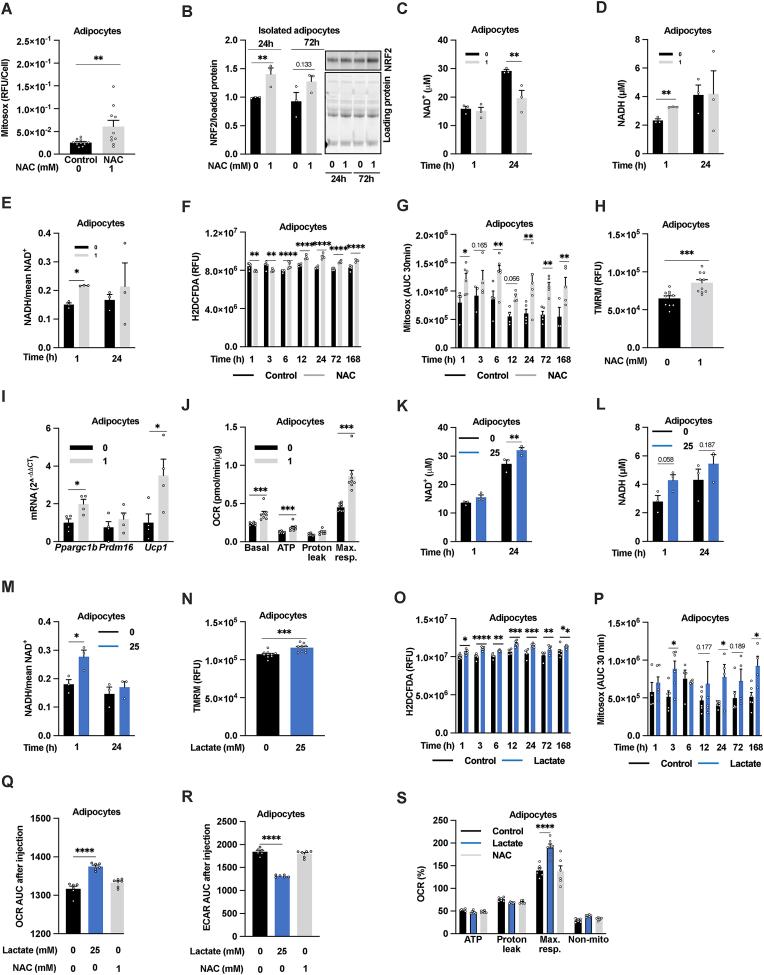
**NAC and lactate, both induce reductive stress associated with time-dependent increase in adipocyte browning**. (A) Mitochondrial ROS production as judged by Mitosox in 3T3-L1 adipocytes pretreated with or without 1 mM NAC for 24h (n = 10/group). (B) NRF2 levels in isolated IWAT adipocytes treated with 1 mM NAC for 24h and 72h (n = 3/group). (C–E) NAD^+^ and NADH levels in 3T3-L1 adipocytes pretreated with or without 1 mM NAC for 1h or 24h (n = 3/group). (F) Total (H2DCFDA) and (G) mitochondrial ROS production (Mitosox) in 3T3-L1 adipocytes pretreated with 1 mM NAC for the indicated periods of time (n = 3–6/group). (H) Mitochondrial membrane potential as judged by tetramethylrhodamine, methyl ester (TMRM) staining in 3T3-L1 adipocytes pretreated with or without 1 mM NAC for 24h (n = 10/group). (I) Relative mRNA expression of *Ppargc1b*, *Prdm16* and *Ucp1* (n = 4/group) and (J) oxygen consumption rate (OCR) in 3T3-L1 adipocytes treated with or without 1 mM NAC for 1 week (n = 10/group). (K–M) NAD^+^ and NADH levels (n = 3/group), (N) mitochondrial membrane potential (TMRM intensity, n = 10/group), (O) total and (P) mitochondrial ROS production in 3T3-L1 adipocytes pretreated with or without 25 mM L-sodium lactate at indicated time points (n = 4–6/group). (Q) OCR and (R) extracellular acidification rate (ECAR) area under the curve measured after acute injection (0–15 min) of 25 mM L-sodium lactate or 1 mM NAC followed by (S) measurements of the OCR with the MitoStress test in 3T3-L1 adipocytes (n = 6/group). *p < 0.05, **p < 0.01, ***p < 0.001, and ****p < 0.0001 for the indicated comparisons.

In accordance with our previous study of 3T3-L1 adipocytes [25], 24h 1 mM NAC treatment induced reductive stress as judged by increased mitochondrial ROS production (Fig. 1A), increased NRF2 nucleus internalization (Suppl. Fig. 1A**),** and NRF2 stabilization in primary IWAT adipocytes **(**Fig. 1B**)**. Moreover, 1h NAC treatment of cultured 3T3-L1 adipocytes was enough to increase NADH levels, the major electron donor for the mitochondrial ETC, thus unbalancing the NADH:NAD^+^ ratio **(**Fig. 1C–E). Notably, NADH levels were like control after 24h NAC treatment, while NAD^+^ levels were lower (Fig. 1C–D). This agrees with the reduced OCR [25] and the increased lactate production at this time point (Suppl. Fig. 1B**)**. Total ROS production was reduced upon short (1 and 3h) NAC treatment but was increased after prolonged (6–168h) treatment **(**Fig. 1F**)**. The mitochondrial ROS production was increased both upon short and longer NAC treatment **(**Fig. 1G**)**. This NAC-induced increase in ROS was associated with mitochondrial hyperpolarization, measured at 24h **(**Fig. 1H**)**. Moreover, the adipocyte *Ppargc1b* and *Ucp1* expression, and OCR were all increased after 1-week-NAC treatment **(**Fig. 1I–J). These findings indicate that high-dose NAC treatment induces a time-dependent mitochondrial response in cultured adipocytes, highlighting NAC as a white adipocyte browning inducer (Suppl. Fig. 1C**)**. A similar, but faster, adipocyte browning effect along with reductive stress is reported to be time- and dose-dependently induced by lactate treatment (24–72h, 5–50 mM) [7]. Lactate is an energy-producing metabolic substrate, which may explain its faster effect on adipocyte browning markers as compared to NAC that is less likely to directly contribute to cellular energy production. Accordingly, we found that 1h 25 mM lactate treatment is sufficient to increase the NADH:NAD^+^ -ratio (Fig. 1K–M). However, this ratio returned to control levels after 24h due to increased NAD^+^ levels (Fig. 1K–M) linked to an increased mitochondrial membrane potential (Fig. 1N), and increased total and mitochondrial ROS-production (Fig. 1O–P). This indicates that the reducing action of lactate is counteracted by increased ETC-mediated recycling of NADH to NAD^+^. Indeed, lactate (25 mM) treatment acutely (within minutes) increased the OCR and reduced the glycolysis rate (ECAR) (Fig. 1Q–R). Lactate treatment also had an acute stimulatory effect on maximal OCR (Fig. 1S). In contrast, acute stimulation with 1 mM NAC was as expected without effect on these parameters (Fig. 1Q–S). We moreover confirmed the previously identified effect of lactate on measures of browning [7] but identified a more complicated dose-response relationship. 10 mM lactate treatment for 24h enhanced the expression *Ppargc1a* and *Ppargc1b* which are key players in mitochondrial biogenesis whilst only a trend for increased *Ucp1* was observed (Suppl. Fig. 1D-F)*.* In contrast, 25 mM lactate induced a 4-5-fold increase in *Ucp1* while 50 mM was without an effect. Both 25 and 50 mM lactate tended to increase *Ppargc1a* and *Ppargc1b,* but the difference compared to control did not reach significance in 3T3-L1 adipocytes (Suppl. Fig. 1D-F).

### 3.2 NRF2 is required for both NAC-and lactate-induced browning in cultured adipocytes

**Fig. 2:**
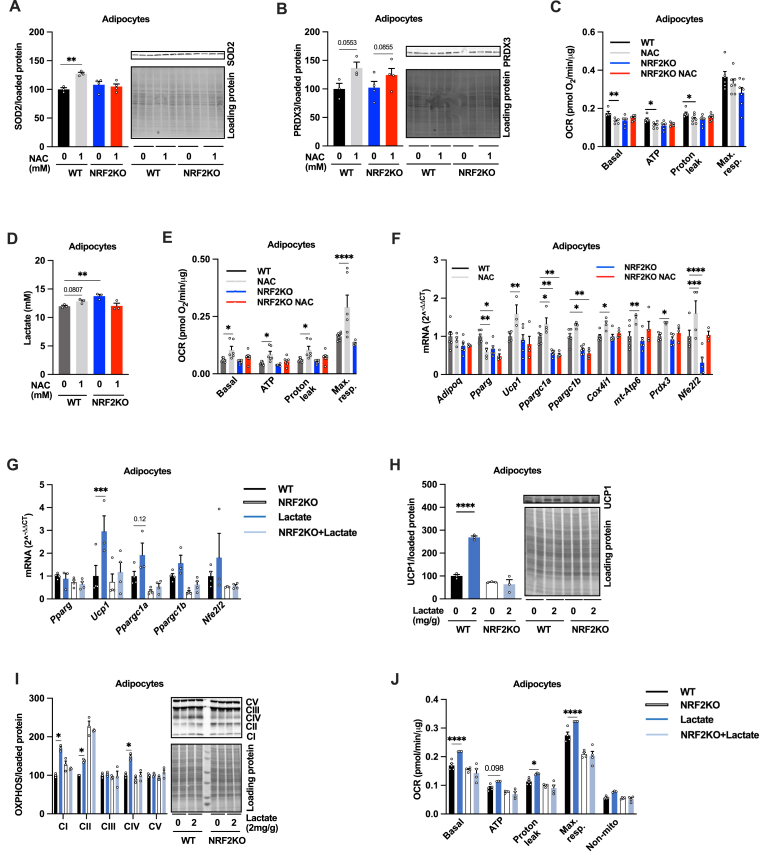
**NRF2 is required for NAC-induced browning in cultured adipocytes**. Effect of 24h 1 mM NAC treatment on (A) SOD2 (n = 3–4/group), (B) PRDX3 (n = 3–4/group), (C) oxygen consumption rate (OCR, n = 6/group), and (D) lactate production (n = 3/group) in adipocytes differentiated from IWAT SVF from adipocyte-specific NRF2 knockout (NRF2KO) and littermate control (WT) mice. Effect of 1-week 1 mM NAC treatment on (E) OCR (n = 6/group) and (F) *Adipoq, Pparg, Ucp1, Ppargc1a, Ppargc1b, cox4i1, mt-Atp6. Prdx3* and *Nfe2I2* levels in adipocytes differentiated from IWAT SVF from adipocyte-specific NRF2KO and WT controls (n = 4–6/group). Effect of 24h 25 mM lactate treatment on (G) *Pparg, Ucp1, Ppargc1a, Ppargc1b,* and *Nfe2I2* levels, (H) UCP1 and (I) electron transport chain protein (OXPHOS) levels, and (J) OCR in adipocytes differentiated from IWAT SVF from adipocyte-specific NRF2KO and WT controls (n = 3–6/group). *p < 0.05, **p < 0.01, ***p < 0.001, and ****p < 0.0001 for the indicated comparisons.

The NRF2 stabilization increases in response to NAC-induced redox stress (Fig. 1B, Suppl. Fig. 1A) as well as in differentiating adipocytes [38]. This adipocyte differentiation associated NRF2 activation is further enhanced when the differentiation is pushed towards beige adipocyte generation (Suppl. Fig. 1G**)**. Accordingly, NRF2 activation has been shown to stimulate mitochondrial function and biogenesis [39,40], and we observe that 24h treatment with the NRF2 activator sulforaphane (SFN, 1 μM) leads to increased expression of antioxidant and mitochondrial function genes such as *Nfe2I2*, *Ucp1*, *Prdx3*, and *Hmox1* **(**Suppl. Fig. 1H**)**. Moreover, 24h SFN treatment decreased maximal OCR while 1-week treatment increased maximal OCR in cultured adipocytes (Suppl. Fig. 1I and J). Based on these observations, we hypothesize that NRF2 at least in part mediates the adipocyte browning effect of both 1-week NAC treatment and 24h lactate treatment. To test this hypothesis, we exploited cultured wildtype (WT) and NRF2 knockout (KO) adipocytes. 24h NAC treatment (1 mM) increased the expression of the mitochondrial antioxidant protein SOD2 in WT but not in the NRF2 KO adipocytes (Fig. 2A). However, the NAC-induced upregulation of PRDX3 was similar between genotypes (Fig. 2B). Moreover, 24h NAC treatment reduced the OCR (basal, ATP-production linked and uncoupled) in WT adipocytes, corroborating our earlier findings [1], while NRF2KO adipocytes displayed reduced OCR and increased lactate production already at baseline and 24h NAC treatment had no additional effect (Fig. 2C–D). As expected from our previous results in 3T3-L1 adipocytes (Fig. 1J), 1-week 1 mM NAC treatment increased OCR (basal, ATP-production-linked, uncoupled, and maximal) and the expression of several browning genes (*Ppargc1a-b*, *Ucp1*, *Prdm16*, *Cox4*, *Apt6*) in the WT adipocytes **(**Fig. 2E–F). However, this effect of NAC treatment was almost completely blunted in NRF2KO adipocytes (Fig. 2E–F). Likewise, 24h 25 mM lactate treatment increased the expression of *Ucp1* and tended to increase the expression of *Ppargc1a* in WT but not in NRF2KO adipocytes (Fig. 2G). This difference was also reflected at the protein and functional level; UCP1 (Fig. 2H), NDUFB8, SDHB and COXII (Fig. 2I) as well as OCR (Fig. 2J) were upregulated by lactate treatment in WT but not in NRF2KO adipocytes. These results indicate that NRF2 is indispensable for the NAC-and lactate-induced browning in cultured adipocytes.

### 3.3 *16-Week high-dose NAC treatment leads to reduced fat mass gain, lower insulin levels and increased IWAT mitochondrial OCR*

The NAC-induced mitohormetic response we observed *in vitro* prompted us to test if such biphasic response to NAC treatment also is in place *in vivo*. To this end, we treated mice with three different NAC doses (0.5, 2 or 10 g/L in drinking water) and analyzed IWAT after 2, 4 and 16 weeks. Mice treated with the highest NAC dose (10 g/L) showed, in line with a previous report [13], reduced weight gain already after 2 weeks (Suppl. Fig. 2A**)**. This difference in weight gain between 10 g/L NAC-treated and control mice increased with time although the food intake was similar (Suppl. Fig. 2B), while the two lower doses had no effect on weight gain (data not shown). The reduced weight gain in the 10 g/L NAC-group after 2 weeks could be explained by a reduction in lean mass gain (Suppl. Fig. 2C). After 4-week NAC treatment, mice receiving the highest NAC dose had increased IWAT levels of the mitochondrial antioxidant PRDX3 (Suppl. Fig. 2D) and *Ppargc1b,* while *Ucp1* levels remained unchanged (Suppl. Fig. 2E).

**Fig. 3:**
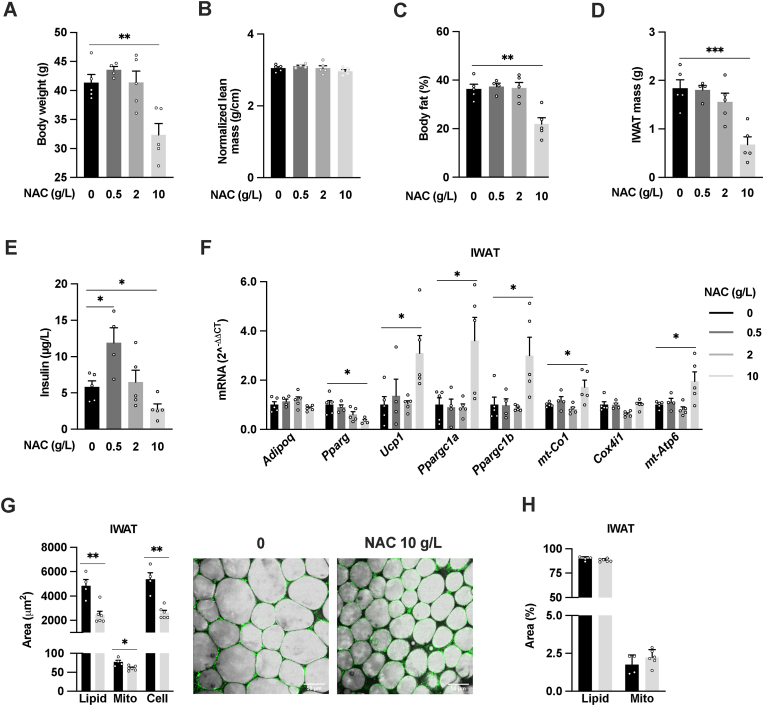
**16-week high-dose NAC treatment leads to reduced fat mass gain, lower insulin levels and increased IWAT mitochondrial OCR**. (A) Body weight, (B) normalized lean body mass, (C) body fat percentage, (D) IWAT mass and (E) serum insulin levels from 22-week-old male mice treated for the last 16 weeks with either regular water or water supplemented with 0.5, 2 or 10 g/L NAC (n = 3–5/group). (F) IWAT relative mRNA expression (*Adipoq, Pparg, Ucp1, Ppargc1a, Ppargc1b, mt-Co1, Cox4i1* and *mt-Atp6*) from 22-week-old male mice treated for the last 16 weeks with either water or NAC 0.5, 2 or 10 g/L solution (n = 3–5/group). (G) Quantification of IWAT adipocyte lipid (grey), mitochondrial (green) and cell area in μm^2^ from CARS and two-photon microscopy, representative images, and (H) lipid and mitochondrial content as % of cell area after 16-week 10 g/l NAC treatment in 22-week-old male (n = 4–6). *p < 0.05, **p < 0.01, ***p < 0.001, and ****p < 0.0001 for the indicated comparisons.

Mice treated with 10 g/L NAC for 16 weeks displayed a 22 % reduction in body weight (Fig. 3A) and a 6 % reduction in body length. Their lean mass was like controls when normalized to length (Fig. 3B). This difference in body weight gain was thus at this time point primarily attributed to a reduction in body fat gain in the 10 g/L NAC group (Fig. 3C). In line with the reduction in body fat gain, the IWAT (Fig. 3D) and gonadal (GWAT) weights (data not shown) were reduced in mice treated with 10 g/L NAC. These changes in body composition in mice treated with 10 g/L NAC were associated with ∼60 % reduced fasting insulin levels. Interestingly, mice treated with 2 g/L NAC showed no change and mice on the lowest (0.5 g/L) NAC dose doubled their fasting insulin levels compared to controls (Fig. 3E). However, the glucose tolerance was equal between groups (Suppl. Fig. 2F).

IWAT from mice treated with 10 g/L NAC for 16 weeks, displayed a marked upregulation of *Ucp1*, *Ppargc1α*, *Ppargc1b*, mt-*Co1* and mt-*Atp6*, suggesting a browning-like response (Fig. 3F). In contrast, *Pparg*, the master regulator of adipocyte differentiation, was downregulated. 16-week-treatment with 0.5 and 2 g/L NAC were without effect on the IWAT expression of these browning markers, suggesting that the proposed mitohormetic process requires a certain level of NAC exposure i.e. 2 g/L may not induce sufficient reductive stress to trigger adaptive responses in adipose tissue. Alternatively, the lower NAC doses would have required a longer treatment period.

To further study the browning-like response after 16-week high-dose NAC treatment, we analyzed adipocyte size and the lipid and mitochondrial content in IWAT. In line with the reduced fat mass gain, high-dose NAC treatment was associated with dramatically reduced adipocyte size and lipid content in IWAT adipocytes, while the mitochondrial content in IWAT adipocytes was slightly (∼20 %) reduced (Fig. 3G). The % lipid and mitochondrial content were however unchanged (Fig. 3H). The OCR in IWAT samples from mice treated with the highest dose was also unchanged and reduced in mice receiving the lowest dose (Suppl. Fig. 2G). However, the mitochondrial content in IWAT is relatively low. Therefore, we analyzed isolated IWAT mitochondria from a new 16-week cohort of controls and highest NAC dose-treated mice. The IWAT mitochondria from mice treated with the highest NAC dose showed increased basal OCR together with a ∼75 % increased ATP production linked-OCR and an almost 2-fold increased uncoupled OCR (Suppl. Fig. 2H). Thus, the percentage of respiration destined to produce ATP was reduced by ∼8 % while proton leak increased by ∼7 % (Suppl. Fig. 2I). In accordance with these OCR data, the ETC proteins were on average 1.8-fold upregulated, and UCP1 was 6-fold upregulated in isolated IWAT mitochondria of the 10 g/l NAC group (Suppl. Fig. 2J and K). These findings show that the IWAT redox and mitochondrial response to NAC treatment *in vivo* is time and dose-dependent; the highest dose triggers redox stress and leads over time to a browning-like response while the lower doses are without effect (Suppl. Fig. 2M).

### 3.4 Adipocyte NRF2 mediates some of the IWAT mitochondrial adaptations to chronic high-dose NAC treatment in mice

**Fig. 4:**
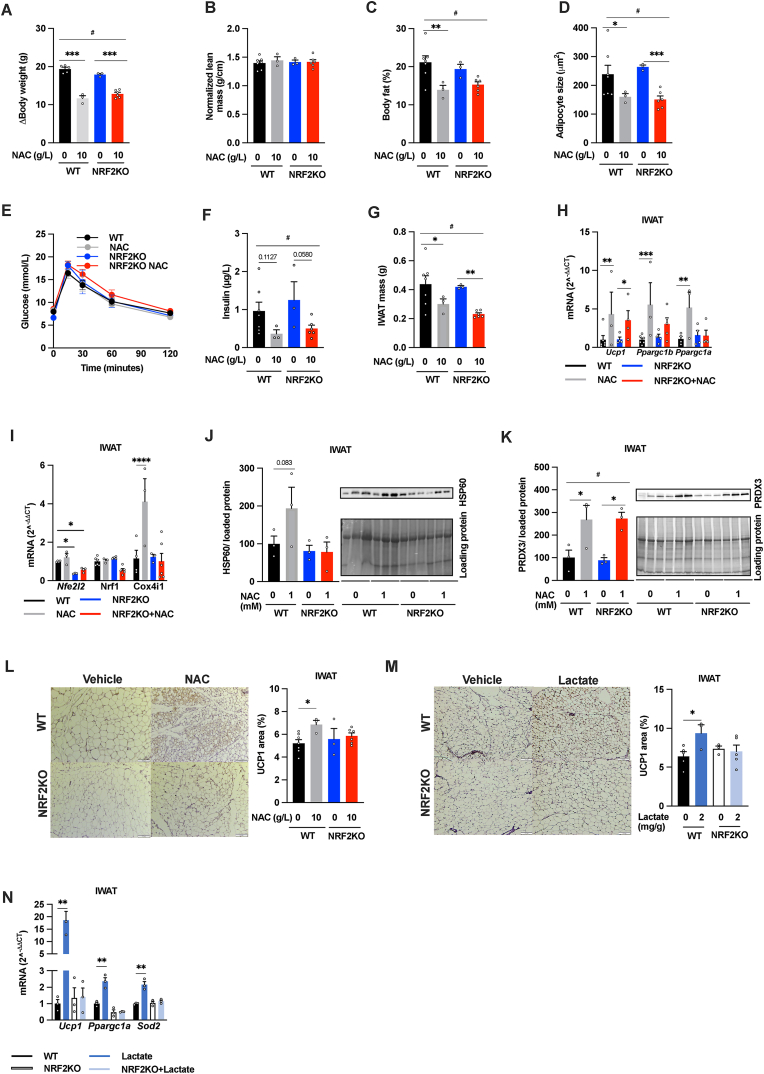
**Adipocyte NRF2 mediates NAC- and lactate-induced IWAT browning**. (A) Body weight gain, (B) normalized lean body mass, (C) body fat percentage, (D) Adipocyte size, (E) oral glucose tolerance test, (F) serum insulin levels, (G) IWAT mass, (H–I) IWAT mRNA levels of *Ucp1, Ppargc1b, Nfe2I2, Nrf1, Ppargc1a and Cox4i1*, (J–K) HSP60, PRDX3 protein levels, and (L) IWAT % UCP1 positive area in 22-week-old male WT and adipocyte-specific NRF2 knockout (NRF2KO) mice treated for the last 16 weeks with either regular water or water supplemented with 10 g/L NAC (n = 3–7/group). (M) Immunostaining and quantification of UCP1 positive areas and (N) *Ucp1, Ppargc1a, Sod2* mRNA levels in IWAT from male 10-11-week-old WT and adipocyte-specific NRF2KO mice treated for the last ten days with daily injections with vehicle or 2 g/kg L-sodium lactate (n = 3–5/group). *p < 0.05, **p < 0.01, ***p < 0.001, and ****p < 0.0001 for the indicated comparisons. For panels A, C, D; F, G, and K; #p < 0.05 for the effect of treatment in 2-way ANOVA.

To test whether NRF2 mediates the IWAT browning-like response in 16-week high-dose (10 g/L) NAC-treated mice, we used adipocyte-specific NRF2 KO mice. In accordance with a previous study [41], adipocyte-specific NRF2 ablation did not affect the body weight gain, body composition, insulin levels, glucose tolerance, IWAT mass and adipocyte size in unchallenged mice (Fig. 4A–G), while the metabolic health was mildly aggravated in HFD-induced obese mice (Suppl. Fig. 3A and B). However, the NAC-induced increase in the mitochondrial markers *Ppargc1a, Ppargc1b*, *Cox41i*, HSP60 and %UCP1 positive area in IWAT was not seen in the adipocyte-specific NRF2 KO mice (Fig. 4H–J, L). In contrast, the NAC-induced reduction in body weight gain, fat percentage, average adipocyte size, insulin levels, IWAT mass, and the upregulation of IWAT PRDX3 and *Ucp1* levels were similar between genotypes (Fig. 4A–H, K). Thus, adipocyte NRF2 contributes to NAC-induced mitochondrial adaptations in white adipocytes *in vivo,* but not to the NAC-mediated changes in adiposity and whole-body metabolic function.

### 3.5 Adipocyte NRF2 mediates lactate-induced browning of IWAT in mice

10-day lactate treatment, which has been shown to induce adipose tissue browning in mice [7], did not alter body weight or body weight change (Suppl. Fig. 3C and D). However, lactate treatment reduced IWAT and GWAT weight in both WT and adipocyte-specific NRF2 KO mice, while the BAT weights were unaffected by treatment or genotype (Suppl. Fig. 3E). Despite the similar IWAT mass in lactate treated mice, only WT mice displayed decreased IWAT adipocyte size (Suppl. Fig. 3F and G) and increased browning as indicated by increased UCP1 positive area (Fig. 4M), and increased expression of *Ucp1*, *Ppargc1a* and *Sod2* (Fig. 4N). Thus, lactate-mediated IWAT browning requires adipocyte NRF2.

### 3.6 HMOX1, downstream of NRF2, is essential for NAC-and lactate-induced browning of cultured adipocytes

**Fig. 5:**
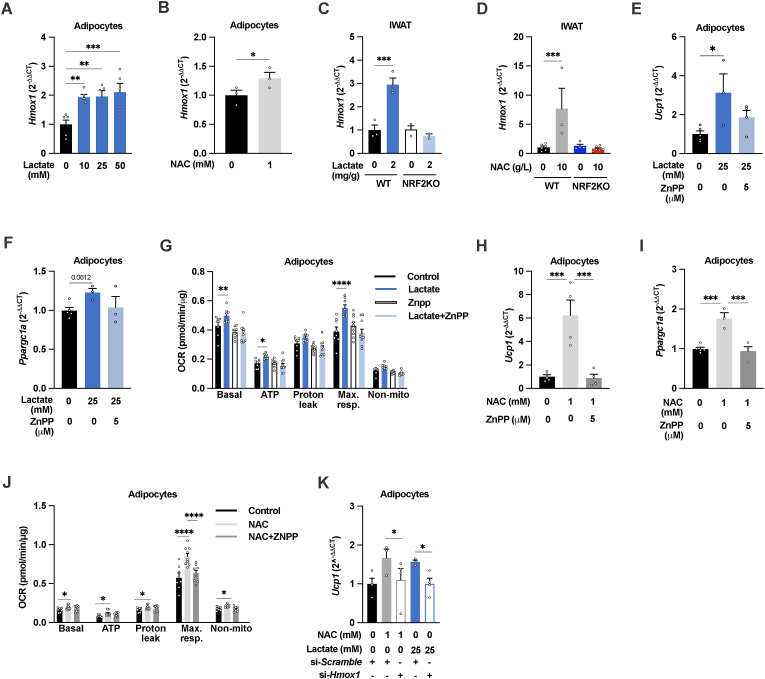
**A NRF2-HMOX1 axis mediates NAC-and lactate-inducedwhite adipocyte browning**. *Hmox1* levels in 3T3-L1 adipocytes treated with (A) 10-, 25- and 50-mM L-sodium lactate (n = 4–6/group) or (B) 1 mM NAC for 24h (n = 3/group). *Hmox1* levels in IWAT from adipocyte-specific NRF2 knockout (NRF2KO) and littermate control mice (WT) treated with (C) 2 g/kg L-sodium lactate for 10 days or (D) 10 g/L NAC for 16 weeks (n = 3–6/group). *Ucp1, Ppargc1a,* and OCR in 3T3-L1 adipocytes treated with (E–G) 25 mM lactate for 24 h, (H–J) 1 mM NAC for 1 week, with or without 5 μM of the HMOX1 inhibitor ZnPP (n = 3–10/group). (K) *Ucp1* levels in 3T3-L1 adipocytes treated with or without 1 mM NAC for 1 week or 25 mM L-sodium lactate for 24h, with or without siRNA-mediated Hmox1 silencing (n = 3–4/group). *p < 0.05, **p < 0.01, ***p < 0.001, and ****p < 0.0001 for the indicated comparisons.

One crucial mediator of the NRF2-mediated cellular response to stress/oxidant is heme oxygenase-1 (HMOX1, also referred to as HO-1) [42]. HMOX1 is a metabolic enzyme critically important for heme degradation that is implicated in excess intracellular iron storage and adipose tissue inflammation in obesity [43,44], but also in adipose tissue browning [45,46]. Indeed, lactate and NAC treatment both, through NRF2, increased the *Hmox1* expression in cultured adipocytes and in IWAT (Fig. 5A–D). In 3T3-L1 adipocytes, chemical inhibition with zinc protoporphyrin (ZnPP, 5 μM) prevented the NAC-, and lactate-induced induction of *Ucp1* and *Ppargc1α* gene expression and increase in OCR (Fig. 5E–J). Similar effects were achieved with siRNA-mediated HMOX1 silencing; ∼25 % reduction in HMOX1 levels (Suppl. Fig. 3H) was associated with lower *Ucp1* expression in 1-week 1 mM NAC- and 24h 25 mM lactate treated adipocytes (Fig. 5K). These data imply that NRF2 through the induction of *Hmox1* and increased HMOX1 activity mediates NAC-and lactate-induced white adipocyte browning.

### 3.7 NRF2 and HMOX1 are required for full IWAT browning effect of chronic β3AR-activation

**Fig. 6:**
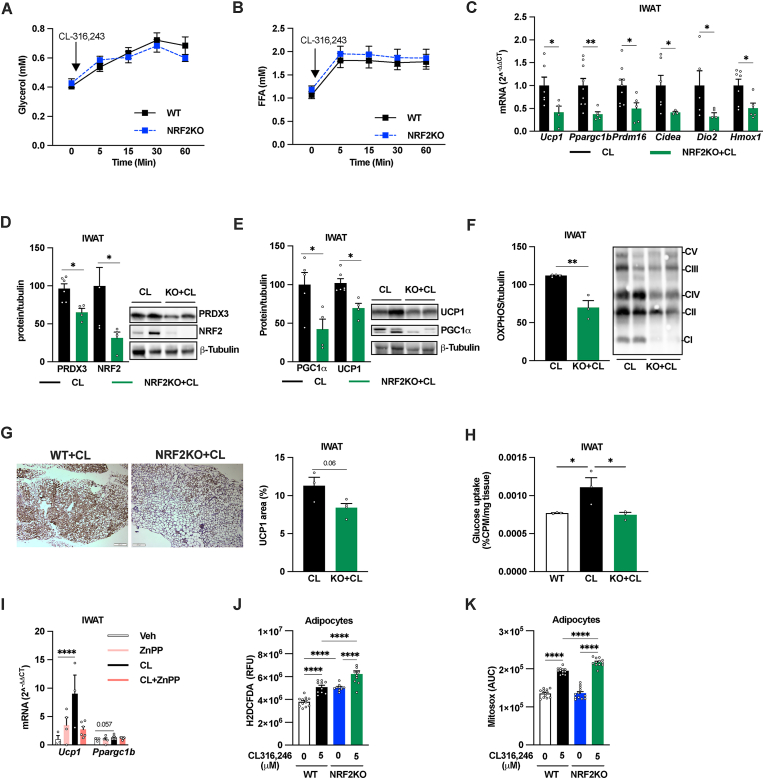
**NRF2 is required for β3-adrenergic receptor activation-induced browning of white adipose tissue**. Serum (A) glycerol and (B) free fatty acid (FFA) levels after injection with the β3 agonist CL-316,243 (1 mg/kg, IP) in male 9-week-old WT and littermate adipocyte-specific NRF2 knockout (NRF2KO) mice (n = 10/group). (C) IWAT levels of *Ucp1, Ppargc1b, Prdm16, Cidea, Dio2* and *Nfe2I2*, and IWAT protein levels of (D) PRDX3, NRF2, (E) UCP1, PGC1α, (F) Electron transport chain protein (OXPHOS), (G) IWAT immunostaining and quantification of UCP1 positive areas, and (H) IWAT glucose uptake in male 10-11-week-old WT and littermate adipocyte-specific NRFKO mice treated daily with CL-316,243 (1 mg/kg, IP) for 10 days (n = 3–7/group). (I) IWAT *Ucp1* and *Ppargc1b* levels in male 8-9-week-old WT mice treated for 10 days with daily IP injections with 1 mg/kg CL-316,243 and/or 250 μg/kg ZnPP (n = 4–6/group). Effect of 1h treatment with 5 μM CL-316,243 on (J) total (H2DCFDA) and (K) mitochondrial ROS production (Mitosox) in adipocytes differentiated from IWAT SVF from adipocyte-specific NRF2KO and littermate WT control (n = 9–12/group). *p < 0.05, **p < 0.01, ***p < 0.001, and ****p < 0.0001 for the indicated comparisons.

To study the possible role of adipocyte NRF2 in “classical” β3AR-mediated adipose tissue browning, we treated WT and adipocyte-specific NRF2KO mice with the β3AR agonist CL-316,243 (CL, 1 mg/kg daily IP injections) for 10 days; a treatment regimen that induces substantial IWAT browning with modest effects on body weight and adiposity in chow-fed lean mice [25,47,48]. While the lipolytic response to CL was similar between genotypes as judged by glycerol and fatty acid levels 15–60 min post injection (Fig. 6A–B), the adipocyte-specific NRF2 KO mice showed a blunted IWAT browning response to chronic CL treatment as judged by reduced *Ucp1*, *Ppargc1b*, *Prdm16*, *Cidea*, *Dio2*, and *Hmox1* levels (Fig. 6C), diminished protein levels of UCP1, PGC1α and ETC proteins (Fig. 6D–F) and a strong trend towards reduced UCP1 positive areas (Fig. 6G). Importantly, the reduction in browning markers was associated with reduced glucose uptake in IWAT of CL-treated adipocyte-specific NRF2KO mice compared to littermate controls (Fig. 6H), suggesting that the altered browning response has functional implications. ZnPP administration reduced the CL-mediated increased in IWAT *Ucp1* (Fig. 6I), indicating that HMOX1 activation is involved also in β3AR-mediated adipose tissue browning. CL-treated mice displayed similar body, IWAT, GWAT, and BAT weights between genotypes as well as between ZnPP and vehicle groups (data not shown).

To further investigate the mechanism between adipocyte NRF2 deficiency and the blunted CL-induced browning response, we measured ROS production in cultured WT and NRF2KO adipocytes with or with CL stimulation. NRF2KO adipocytes showed higher total ROS production in both unchallenged and CL-stimulated conditions (Fig. 6J). Moreover, the CL-induced increase in mitochondrial ROS production was enhanced in NRF2KO adipocytes while the basal mitochondrial ROS production was similar between genotypes (Fig. 6K)**.**

## 4 Discussion

Previous research highlight ROS and oxidative stress as important regulators of adipocyte and mitochondrial function [49]. This study adds further mechanistic insight within this area and underscore the significance of NRF2 in adipocyte functionality, in particular within the context of reductive stress induced by NAC or lactate but also in β3AR-mediated adipose tissue browning.

### 4.1 Effects of NAC on metabolic health and adipose tissue browning and the role of adipocyte NRF2

Many studies have used antioxidants as therapeutic and experimental tools to reduce oxidative stress. *In vivo* [12,13,16,50,51] and *in vitro* studies of myocytes [52] and tumor cells [53] have studied the effects of NAC using a broad range of concentrations and duration, from 2 weeks to 4 months, showing effects on multiple biological processes and describing both beneficial and deleterious effects. However, the mechanism for these seemingly contradictory effects of NAC on metabolic readouts is understudied. Mice exposed to NAC for 4 weeks displayed a dose-dependent elevation of PRDX3 and *Ppargc1b* in IWAT. These data are in line with the observed NAC-induced increase in mitochondrial ROS production (that should elevate PRDX3 [25,54]) and mitohormesis [55] in cultured white adipocytes. However, we acknowledge that one limitation of our study is the absence of direct measurements of mitochondrial ROS or reductive stress dynamics *in vivo*. After 16-weeks of NAC treatment, mice receiving the highest dose displayed reduced fat mass gain, reduced insulin levels, reduced adipocyte size, and IWAT browning. In contrast, mice treated with lowest dose NAC displayed reduced IWAT OCR and increased insulin levels. These data are accordance with a dose-dependent mitohormetic process, although the link between IWAT mitochondrial function and insulin levels may not be causal. Nevertheless, our results from high dose NAC treatment are in line with previous reports showing that NAC treatment improves the insulin sensitivity in hyperinsulinemic patients [51], in insulin-resistant rats [56] and in HFD-fed mice [16]. In all these studies, the reversal of insulin resistance was attributed to NAC's antioxidant properties and the consequent anti-inflammatory effect. However, we believe our data open for alternative mechanism(s) for the metabolic improvement in NAC-treated animals and patients. *In vitro* and *in vivo*, we found that NAC-induced white adipocyte browning is largely dependent on NRF2. In sharp contrast, adipocyte NRF2 had no impact on NAC-induced changes in body weight, adiposity, and insulin levels. This is not surprising as the reduced weight gain in high-dose NAC-treated mice is apparent immediately while the increased IWAT thermogenesis is only detected in isolated mitochondria and not observed until many weeks of treatment. There are several plausible explanations for these adipocyte NRF2-independent effects. For instance, NAC will be converted into l-cysteine that at high concentrations can reduce appetite and food intake [57]; effects that are associated with reduced weight and fat mass gain in rats [58]. However, our data suggest that other mechanisms than a difference in food intake are at play. It is possible that the NAC metabolite S-nitro-N-acetylcysteine induce vasodilation peripherally [50,59] and that a high dose of NAC induces reductive stress (and NRF2) in various cell types including centrally leading to increased SNS outflow [60] and thereby slower weight gain, increased lipolysis, and increased energy expenditure. Moreover, NAC may also affect adipose tissue expansion directly as suggested by *in vitro* studies showing dose-dependent inhibition of adipogenesis and adipocyte lipid accumulation [61]. We also acknowledge that NAC-mediated systemic effects originate from other yet unknown mechanisms.

### 4.2 Adipocyte NRF2 mediates adaptive browning processes in white adipose tissue

Lactate is generated when the glycolytic flux exceeds mitochondrial oxidative capacities [62]. Adipocytes produce high levels of lactate [63], and this production is further stimulated by NAC treatment (this study and [25]). Lactate is considered a redox signaling metabolite that shuttles to other cells and tissues to be oxidized [64] e.g. adipocyte lactate increases glucose-mediated lipogenesis in cancer cells [63]. Lactate is also reported to alleviate the intracellular redox state by inducing adipocyte browning [7,65]. During this process lactate is imported into the mitochondria by the Monocarboxylate transporter 1 [66], which unbalances the lactate:pyruvate concentration, switching the glycolytic enzyme lactate dehydrogenase to oxidize lactate to pyruvate along with reduction of NAD^+^ to NADH. Notably, recent research suggests that the browning-inducing effect of lactate largely can be attributed osmotic overloading by its counterion sodium [67]; an effect we did not control for. Nevertheless, our *in vitro* and *in vivo* data show that, in states of mitochondrial stress, adipocyte NRF2 is necessary for the adaptive increase of UCP1 that allows excess NADH to be oxidized by the ETC complex I [10]. We also found that adipocyte NRF2 plays a significant role in the more pronounced browning response triggered by chronic β3AR agonist treatment as evident from a >50 % decrease in IWAT browning markers along with reduced IWAT glucose uptake in adipocyte-specific NRF2 KO mice. However, the acute lipolytic response to β3AR activation was similar between genotypes, which may explain the comparable fat pad weights. These data are in line with the blunted β3AR agonist-induced increase in *Ucp1* in isolated NRF2 KO adipocytes [68], and suggest the existence of a β3AR-NRF2-UCP1 axis controlling both redox homeostasis and thermogenesis in white adipocytes.

### 4.3 NRF2, HMOX1 and white adipose tissue browning

HMOX1 catalyzes the rate-limiting step of heme oxidation to biliverdin, carbon monoxide, and free ferrous iron [42]. Changes in the cellular iron amount affect the dynamics of cellular and whole-body lipid homeostasis. For example reduction in mitoNEET, an integral iron-sulfur (FeS)-containing protein that regulates mitochondrial iron content [69], enhances mitochondrial respiratory capacity, but also causes heightened oxidative stress and glucose intolerance despite reduced weight gain [70]. HMOX1 is regulated at the transcriptional levels and can be induced by NRF2-activation and by a wide range of drugs and naturally occurring compounds and elements [47]. The NRF2-HMOX1 pathway has previously been implicated in adipocyte browning *in vitro* [46], and adipocyte-specific HMOX1 overexpression attenuates HFD-induced adiposity and metabolic dysfunction in mice [71]. Here, we found that NAC, lactate, and β3AR agonist treatment, all increased *Hmox1* levels in cultured adipocytes and/or in IWAT. This *Hmox1* induction was dependent on adipocyte NRF2. Furthermore, HMOX1 inhibition suppressed both NAC- and lactate-induced browning *in vitro* and β3AR agonist-induced browning *in vivo*. Thus, our results indicate that mitochondrial iron regulation, through NRF2-HMOX1, is central to redox-stress-induced white adipose tissue browning. In addition, the blunted browning response in NRF2 deficient adipocytes can also be considered as an adaptive response to prevent further increase in ROS production.

## Conclusions

Our results provide an alternative explanation to the current paradigm where antioxidants primarily are thought to act as scavengers and thereby improve health through ROS neutralization. High-dose NAC or lactate treatment to unchallenged chow-fed mice or cultured adipocytes induces reductive stress leading to browning via activation of the NRF2-HMOX1-pathway. This mechanism is also involved in conventional white adipose tissue browning induced by β3AR-activation, and may thus deliver therapeutic targets for the prevention or treatment of obesity-associated metabolic disorders.

## Declaration of competing interest

The authors declare that they have no known competing financial interests or personal relationships that could have appeared to influence the work reported in this paper.

## Data Availability

Data will be made available on request.
