# Supplementary material for: NRF2 is essential for adaptative browning of white adipocytes
Source: Redox Biol. 2023 Oct 31;68:102951. doi: 10.1016/j.redox.2023.102951 (PMC10652207; doi:10.1016/j.redox.2023.102951)
Supplement: Multimedia component 1 [file mmc1.pdf]

Supplemental Table 1.

Gene	Forward primer (5’-3’)	Reverse primer (5’-3’)
Actb	GACCCAGATCATGTTTGAGA	GAGCATAGCCCTCGTAGAT
Adipoq	TGTTCCTCTTAATCCTGCCCA	TGTTCCTCTTAATCCTGCCCA
Cidea	TGCTCTTCTGTATCGCCCAGT	GCCGTGTTAAGGAATCTGCTG
Cox4i1	CATACTTTCGATCGTGACTGGGTG	TTCTTGTCATAGTCCCACTTGGCG
Dio2	TGTCTGGAACAGCTTCCTCC	CCATCAGCGGTCTTCTCCG
Gapdh	ATGGCCTTCCGTGTTCCTAC	GCCTGCTTCACCACCTTCTT
Homx1	TGTGAGGGACTCTGGTCTTTGTG	GAACACTCTGGAGATGACACCTGAA
mt-Atp6	ACTTGCCCACTTCCTTCCACA	TAAGCCGGACTGCTAATGCCA
mt-Co1	CAATGGGAGCAGTGTTTGCT	ATATGATGGCGAAGIGGGCT
Nfe2l2	AGGTGACGAGATGGGCTGC	CGTTGTCCCCATTTTTGCG
Nrf1	ACCTTTGGAGAATGTGGTGCGA	TGAATTAACCTCCTGTGGCCGA
Pparg	TATGGAGTGACATAGAGTGTGCT	CCACTTCAATCCACCCAGAAAG
Ppargc1a	ACAGCTTTCTGGGTGGATTG	TGAGGACCGCTAGCAAGTTT
Ppargc1b	TCCTGTAAAAGCCCGGAGTAT	GCTCTGGTAGGGGCAGTGA
Prdm16	GCAGACCCTGTGGGAGTCCTGAAA	GCTCCCCTGTGTGTGTCCTCAGAT
Prdx3	AACACACCAAGAAAGAATGGTGG	GACACTCAGGTGCTTGACG
Sod2	CCAGTGCAGGACCTCATTTT	CACCTTTGCCCAAGTCATCT
Ucp1	GTGAAGGTCAGAATGCAAGC	AGGGCCCCCTTCATGAGGTC

Suppl. Fig.1

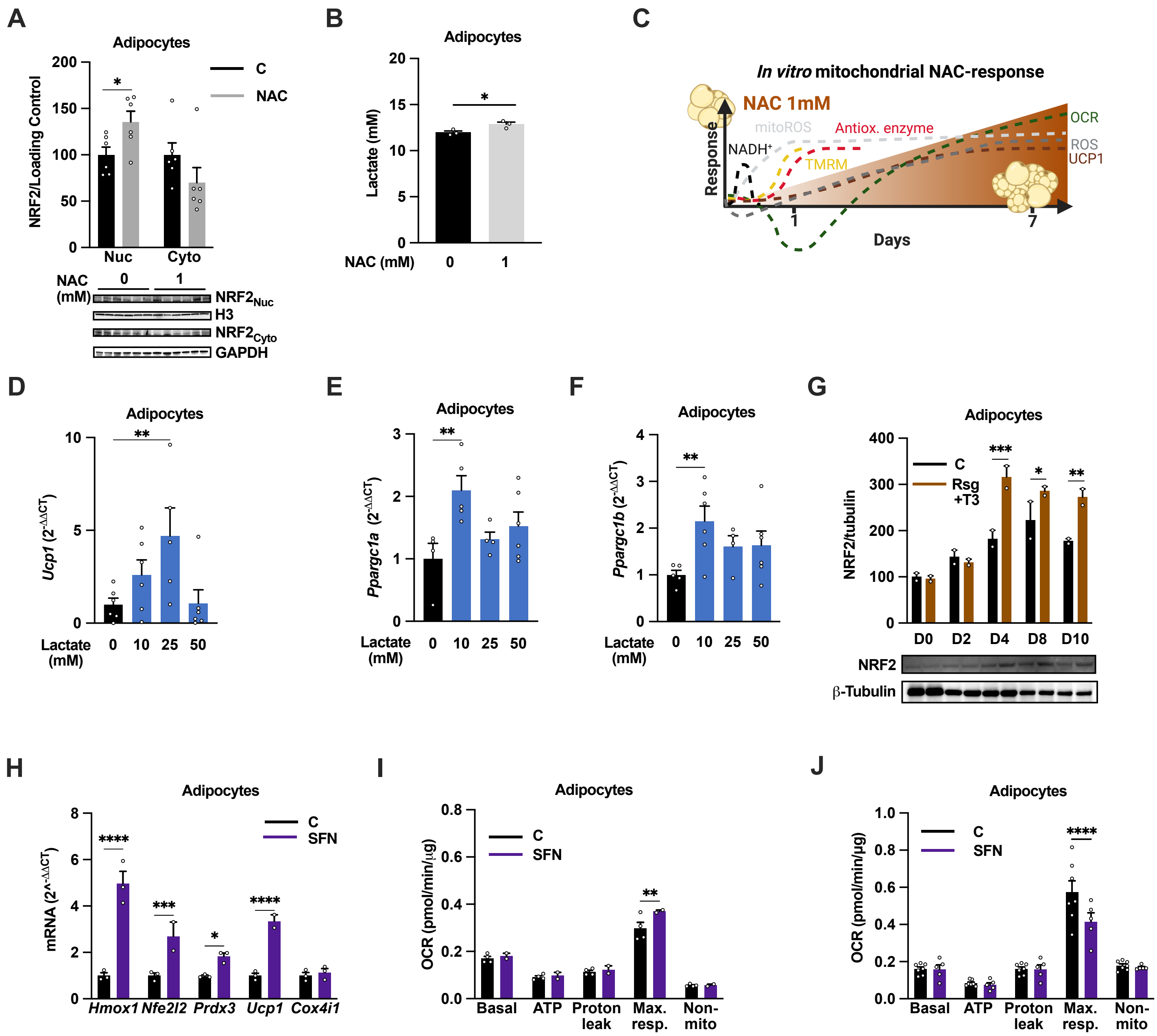

A
Adipocytes
NRF2/Loading Control
C
NAC
Nuc
Cyto
NAC (mM)
0
1
NRF2Nuc
H3
NRF2Cyto
GAPDH
B
Adipocytes
Lactate (mM)
NAC (mM)
C
In vitro mitochondrial NAC-response
NAC 1mM
mitoROS
Antiox. enzyme
NADH+
TMRM
OCR
ROS
UCP1
Response
Days
D
Adipocytes
Ucp1 (2-ΔΔCT)
Lactate (mM)
E
Adipocytes
Ppargc1a (2-ΔΔCT)
Lactate (mM)
F
Adipocytes
Ppargc1b (2-ΔΔCT)
Lactate (mM)
G
Adipocytes
NRF2/tubulin
C
Rsg +T3
D0
D2
D4
D8
D10
NRF2
β-Tubulin
H
Adipocytes
mRNA (2^-ΔΔCT)
C
SFN
Hmox1
Nfe2l2
Prdx3
Ucp1
Cox4i1
I
Adipocytes
OCR (pmol/min/µg)
C
SFN
Basal
ATP
Proton leak
Max. resp.
Non-mito
J
Adipocytes
OCR (pmol/min/µg)
C
SFN
Basal
ATP
Proton leak
Max. resp.
Non-mito

Suppl. Fig.2

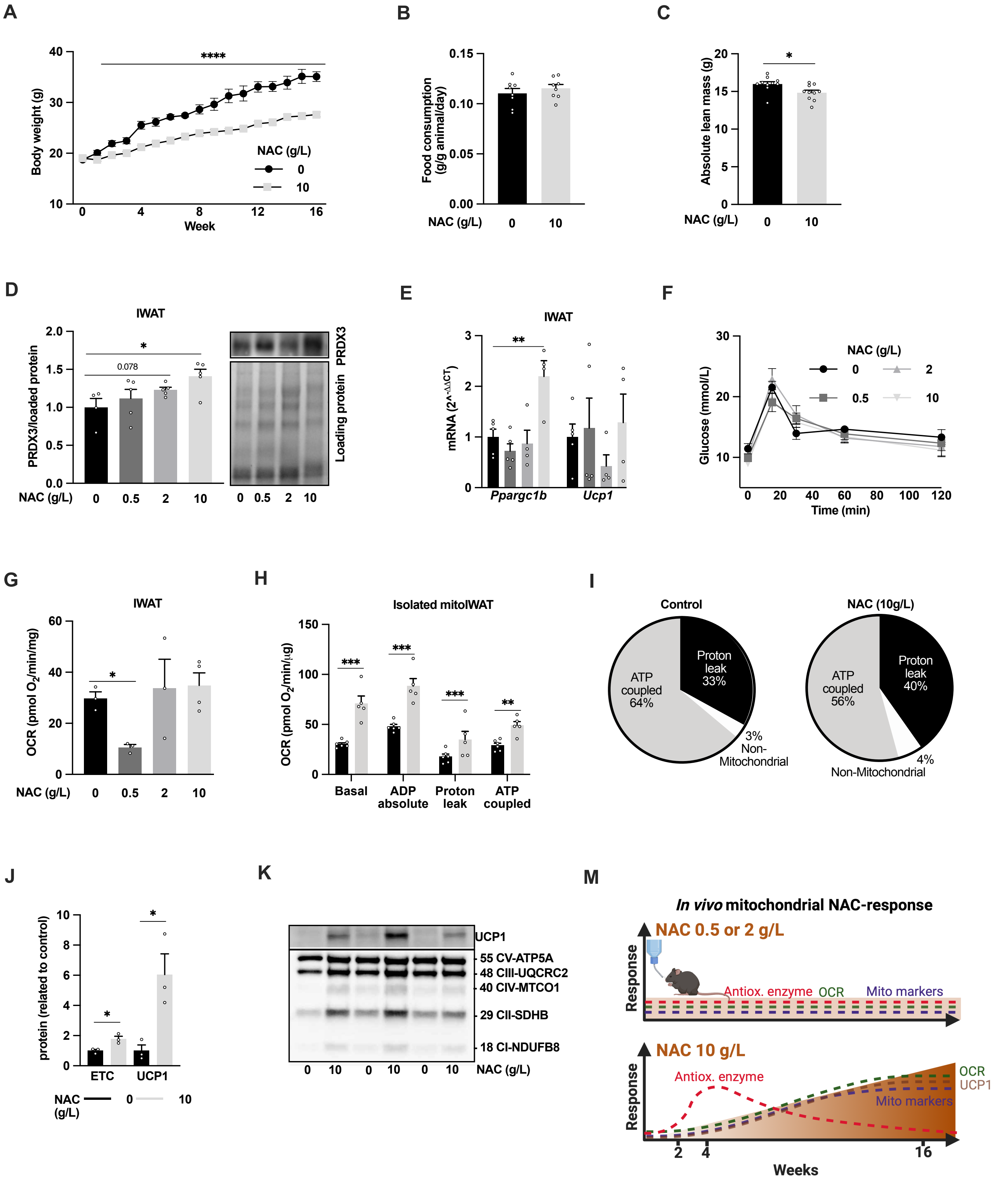
A
Body weight (g)
Week
NAC (g/L)
0
10
****
B
Food consumption (g/g animal/day)
NAC (g/L)
C
Absolute lean mass (g)
*
D
IWAT
PRDX3/loaded protein
0.078
*
PRDX3
Loading protein
NAC (g/L) 0 0.5 2 10
E
IWAT
mRNA (2^-ΔΔCT)
**
Ppargc1b
Ucp1
F
Glucose (mmol/L)
Time (min)
NAC (g/L)
0 2
0.5 10
G
IWAT
OCR (pmol O2/min/mg)
*
H
Isolated mitoIWAT
OCR (pmol O2/min/µg)
***
**
Basal
ADP absolute
Proton leak
ATP coupled
I
Control
NAC (10g/L)
Proton leak 33%
ATP coupled 64%
3% Non-Mitochondrial
Proton leak 40%
ATP coupled 56%
4% Non-Mitochondrial
J
protein (related to control)
ETC
UCP1
NAC (g/L) 0 10
K
UCP1
55 CV-ATP5A
48 CIII-UQCRC2
40 CIV-MTCO1
29 CII-SDHB
18 CI-NDUFB8
0 10 0 10 0 10 NAC (g/L)
M
In vivo mitochondrial NAC-response
NAC 0.5 or 2 g/L
Response
Antiox. enzyme OCR Mito markers
NAC 10 g/L
Antiox. enzyme
OCR
UCP1
Mito markers
2 4 16
Weeks

Suppl. Fig.3

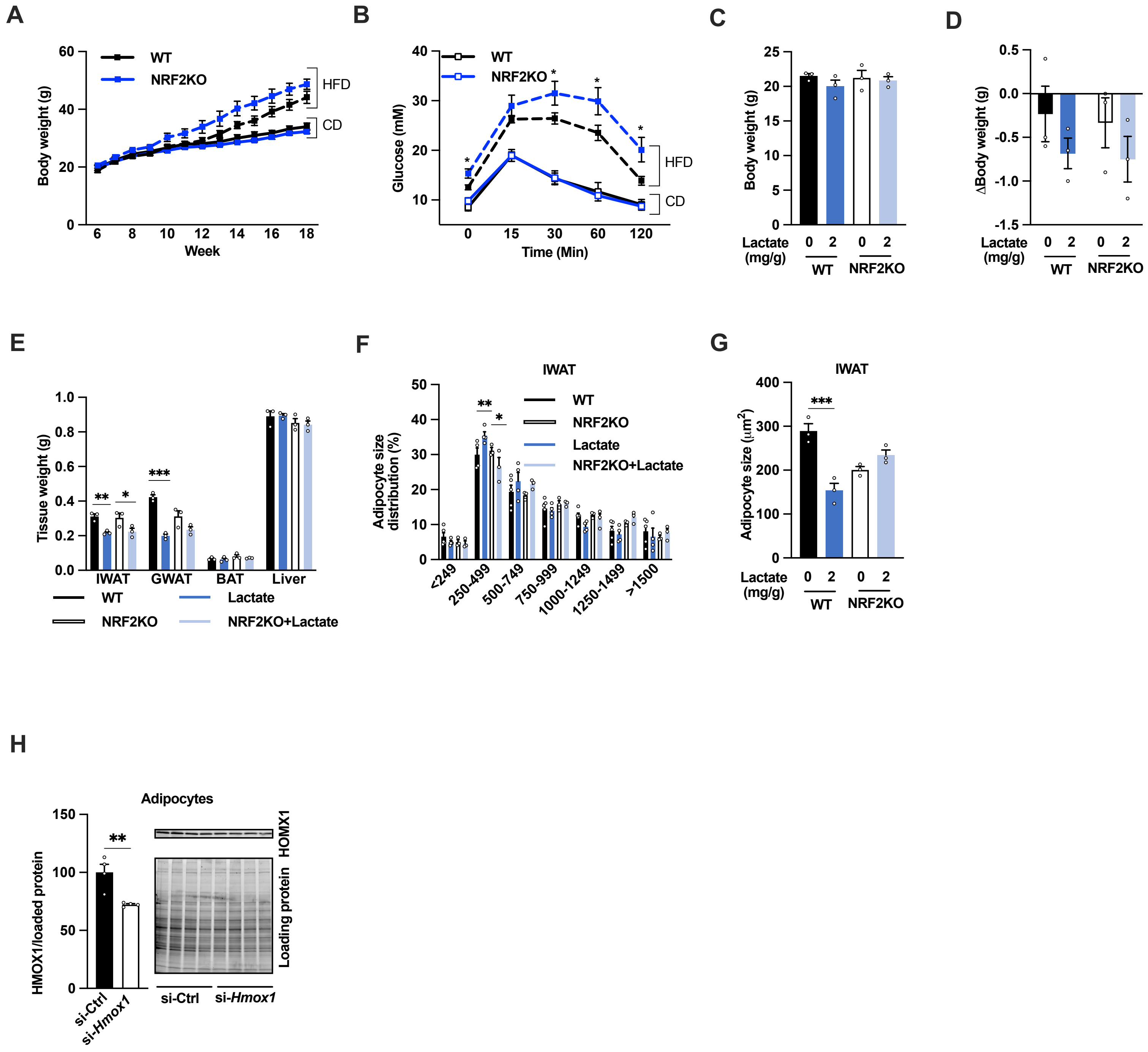

A
Body weight (g)
WT
NRF2KO
HFD
CD
Week
B
Glucose (mM)
WT
NRF2KO
HFD
CD
Time (Min)
C
Body weight (g)
Lactate (mg/g)
WT
NRF2KO
D
ΔBody weight (g)
Lactate (mg/g)
WT
NRF2KO
E
Tissue weight (g)
IWAT
GWAT
BAT
Liver
WT
Lactate
NRF2KO
NRF2KO+Lactate
F
IWAT
Adipocyte size distribution (%)
WT
NRF2KO
Lactate
NRF2KO+Lactate
<249
250-499
500-749
750-999
1000-1249
1250-1499
>1500
G
IWAT
Adipocyte size (μm2)
Lactate (mg/g)
WT
NRF2KO
H
Adipocytes
HMOX1/loaded protein
HOMX1
Loading protein
si-Ctrl
si-Hmox1

Supplemental Figure 1. Effects of lactate and sulforaphane treatment on mitochondrial function markers in adipocytes. (**A**) NRF2 translocation in 3T3-L1 adipocytes treated with or without 1 mM NAC for 24h (n=6/group). (**B**) Lactate production in adipocytes differentiated from IWAT stromal vascular fraction treated with or without 1 mM NAC for 24h (n=3/group). (**C**) Representative scheme of time-dependent NAC response in cultured adipocytes treated with 1mM NAC. Relative levels of **(D**) *Ucp1,* (**E**) *Ppargc1a* and (**F**) *Ppargc1b* in 3T3-L1 adipocytes pretreated with or without 25 mM lactate for 24h (n=4-6/group). (**G**) Relative protein levels of NRF2 during adipocyte differentiation of 3T3-L1 cells with or without the addition of Rosiglitazone (1 μM) and triiodothyronine (T3, 50 nM) to induce beige-like adipocytes (n=2/group). (**H**) Relative levels of *Hmox1, Nfe2l2, Prdx3, Ucp1, Cox4l1* and (**I**) oxygen consumption rate (OCR) in 3T3-L1 adipocytes treated with or without 1μM Sulforaphane (SFN) for 24h (n=2-4/group). (**J**) OCR in adipocytes differentiated from IWAT stromal vascular fraction treated with or without 1 μM SFN for 1 week (n=5-7/group). *p<0.05, **p<0.01, ***p<0.001, and ****p<0.0001 for the indicated comparisons.

Supplemental Figure 2. High-dose NAC treatment leads to reduced weight gain and increased OCR in IWAT mitochondria. (**A**) Growth curves of mice treated with either water or water supplemented with 10 g/L NAC for 16 weeks (n=10/group). (**B**) Average daily food consumption and (**C**) lean body mass in mice treated with either water or water supplemented with 10 g/L NAC for 2 weeks (n=8-10/group). (**D**) Relative PRDX3 levels in IWAT, (**E**) relative *Pgc1β* and *Ucp1* levels in IWAT after 4-week treatment with regular or NAC-supplemented drinking water (n=4-5/group). (**F**) Oral glucose tolerance and (**G**) IWAT OCR in mice treated with regular or NAC-supplemented drinking water for 16 weeks (n=3-5/group). (**H**) Basal respiration and OCR after the addition of ADP ("ADP absolute"), proton leak and ATP-linked OCR expressed as pmol/min/g mitochondrial protein and as (**I**) % of ADP absolute of isolated mitochondria from IWAT samples from mice treated with or without 10 g/l NAC-supplemented drinking water for 16 weeks (n=3-5/group). (**J-K**) Quantification and blot of electron transport chain (ETC) and UCP1 protein from isolated

mitochondria from IWAT samples from mice treated with or without 10 g/l NAC-supplemented drinking water for 16 weeks (n=3/group). (**M**) Representative scheme of time- and dose-dependent NAC response in IWAT of mice treated with 0.5, 2 or 10 g/l NAC-supplemented drinking water. All mice were male and six weeks old at the treatment start. *p<0.05, **p<0.01, ***p<0.001, and ****p<0.0001 for the indicated comparisons.

Supplemental Figure 3. NRF2 is required for lactate-induced browning in adipocytes. (**A**) Growth curves and (**B**) oral glucose tolerance in 18-week-old WT and littermate adipocyte-specific NRF2 knockout (NRF2KO) male mice fed either chow (CD) or high fat diet (HFD) for the last 12 weeks (n=3/group). (**C**) Body weight, (**D**) body weight change, (**E**) IWAT, GWAT, BAT and liver mass, and IWAT (**F**) adipocyte size distribution and (**G**) average size in WT and littermate adipocyte-specific NRF2KO mice treated with 2g/kg lactate IP once per day for ten days (n=3-4/ group). (**H**) Relative HMOX1 levels in 3T3-L1 adipocytes treated with 5 nM of Hmox-1 (si-Hmox1) or Scramble (si-Control) siRNA (n=4/group). *p<0.05, **p<0.01, and ***p<0.001 for the indicated comparisons.
